# Supplementary material for: Public Perceptions of Contentious Risk: The Case of Rubber Granulate in the Netherlands
Source: Int J Environ Res Public Health. 2019 Jun 25;16(12):2250. doi: 10.3390/ijerph16122250 (PMC6616659; doi:10.3390/ijerph16122250)
Supplement: Supplementary file 1 [file ijerph-16-02250-s001.pdf]

Supplementary Materials

Table S1. Pearson's correlations between all dependent variables at T1.

	N T1	Correlations T1*					
		(1)	(2)	(3)	(4)	(5)	(6)
Nature of the hazard (1)	1033	-					
Exposure (2)	1005	0.46	-				
Possible health effects (3)	1033	0.65	0.51	-			
Probability of health effects (4)	1033	0.56	0.54	0.75	-		
Negative affective response (5)	1033	0.59	0.44	0.60	0.63	-	
Regulatory mitigation preferences (6)	1033	0.49	0.39	0.50	0.47	0.54	-
Strict mitigation preferences (7)	1033	0.57	0.49	0.60	0.64	0.66	0.58

* All correlations are significant at the 0.01 level (two-tailed)

Table S2. Pearson's correlations between all dependent variables at T2

	N T2	Correlations T2*					
		(1)	(2)	(3)	(4)	(5)	(6)
Nature of the hazard (1)	782	-					
Exposure (2)	773	0.41	-				
Possible health effects (3)	782	0.65	0.52	-			
Probability of health effects (4)	782	0.55	0.53	0.75	-		
Negative affective response (5)	782	0.61	0.43	0.66	0.65	-	
Regulatory mitigation preferences (6)	782	0.53	0.40	0.58	0.52	0.54	-
Strict mitigation preferences (7)	782	0.60	0.50	0.72	0.71	0.71	0.65

* All correlations are significant at the 0.01 level (two-tailed)
